# Intermittent Hypoxia after Transient Focal Ischemia Induces Hippocampal Neurogenesis and c-Fos Expression and Reverses Spatial Memory Deficits in Rats

**DOI:** 10.1371/journal.pone.0024001

**Published:** 2011-08-24

**Authors:** Yi-Wei Tsai, Yea-Ru Yang, Paulus S. Wang, Ray-Yau Wang

**Affiliations:** 1 Department and Institute of Physical Therapy and Assistive Technology, National Yang-Ming University, Taipei, Taiwan; 2 Department and Institute of Physiology, National Yang-Ming University, Taipei, Taiwan; University of Memphis, United States of America

## Abstract

**Background:**

Memory impairment is a frequent complication of brain ischemia. Neurogenesis is implicated in learning and memory and is regulated by the transcription factor c-Fos. Preconditioning intermittent hypoxia (IH) attenuates ischemia-related memory impairments, but it is not known whether post-ischemia IH intervention has a similar effect. We investigated the effects of post-ischemia IH on hippocampal neurogenesis and c-Fos expression as well as spatial learning and memory in rats.

**Methodology/Principal Findings:**

Focal cerebral ischemia was induced in some rats by middle cerebral artery occlusion (MCAO), while other rats received sham MCAO surgery. Beginning a week later, half of the rats of each group received IH interventions (12% oxygen concentration, 4 hrs/d, for 7 d) and half received sham IH sessions. An additional group of rats received MCAO, IH, and injections of the neurogenesis-impairing agent 3′-AZT. Spatial learning and memory was measured in the Morris water maze, and hippocampal neurogenesis and c-Fos expression were examined. Hypoxia-inducible factor 1α (HIF-1α) and phosphorylated mitogen-activated protein kinase (pMAPK) were considered as possible mediators of IH-induced changes in neurogenesis and c-Fos expression. IH intervention following MCAO resulted in recovered spatial memory, increased hippocampal neurogenesis, and increased expression of c-Fos in newborn hippocampal cells. These effects were blocked by 3′-AZT. IH intervention following MCAO also was associated with increased hippocampal pMAPK and HIF-1α expression.

**Conclusions/Significance:**

IH intervention following MCAO rescued ischemia-induced spatial learning and memory impairments, likely by inducing hippocampal neurogenesis and c-Fos expression through mediators including pMAPK and HIF-1α

## Introduction

Memory formation, maintenance, and retrieval are dynamic processes involving transcription, translation, and expression of protein [1]. The transcription factor c-Fos is strongly implicated in memory formation. Experience and spatial learning stimulate c-Fos expression [2], and *c-fos* knockout mice exhibit deficits in long-term memory and synaptic plasticity [3]. Moreover, memory impairment following brain ischemia often is associated with decreased c-Fos expression [4], [5], [6]. Therefore, transcription factors such as c-Fos can be used as memory markers.

Neurogenesis is the birth of new neurons in the adult mammalian brain, predominantly in the subventricular zone and the hippocampal dentate gyrus (DG) [7]. Inhibiting neurogenesis in the DG impairs learning and memory in animals [8], and environmental enrichment is associated with facilitated neurogenesis and increased expression of c-Fos in the DG [9]. Hence, newborn neurons in the DG express transcription factors related to memory formation.

Preconditioning intermittent hypoxia (IH) attenuates brain damage [10] and learning and memory impairments observed following severe ischemia [11], [12]. These effects may be related to IH-induced neurogenesis in the DG [13] and increased expression of transcription factors such as c-Fos [11]. However, it is not clear whether IH administered post-ischemia can facilitate neurogenesis, stimulate c-Fos expression, or ameliorate learning and memory deficits. The purpose of the present study was to investigate the effects of IH intervention following brain ischemia in rats.

## Results

### Spatial learning and memory impairments following ischemia are reversed by post-ischemia IH intervention

**Figure 1:**
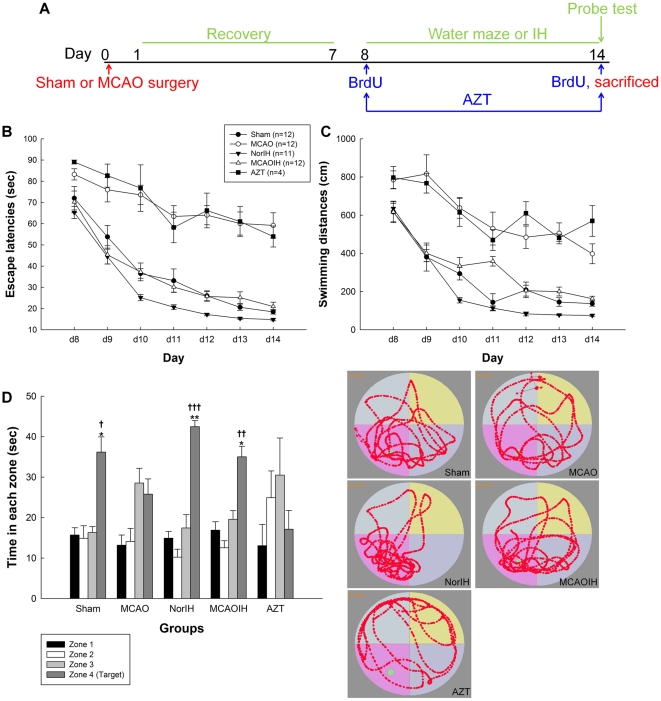
Reversal of spatial learning and memory impairments in ischemic rats following IH intervention. (A) Experimental design. (B) Escape latencies observed during 7 consecutive days of training on the MWM. (C) Swimming distances seen during 7 consecutive days of training on the MWM. (D, left) Time spent in the target quadrant (zone IV) during the MWM probe test. (D, right) Swim paths taken by a representative rat from each group during the MWM probe test. *p<0.05, **p<0.01, compared to the MCAO group. †*p*<0.05, ††*p*<0.01, †††*p*<0.001, compared to the AZT group.

Separate groups of rats were exposed to middle cerebral artery occlusion (MCAO) or sham MCAO surgery, followed one week later by 7 d of IH intervention or sham IH treatment, as described in the Materials and Methods. To investigate the effect of IH on spatial learning and memory following MCAO, rats were trained and tested on the Morris Water Maze (MWM) on each of the 7 d of IH intervention or sham IH treatment (Days 8–14; Figure 1A).

Following ischemia (MCAO group), rats exhibited markedly decreased spatial learning abilities as indicated by both escape latency and distance traveled prior to locating the platform (MCAO vs. Sham, escape latency, *p*<0.001; swimming distance, *p*<0.001; Figure 1B–1C). However, rats exposed to IH intervention after ischemia (MCAOIH group) not only performed significantly better than rats receiving sham IH treatment after ischemia (MCAOIH vs. MCAO, escape latency, *p*<0.001; swimming distance, *p*<0.001), but performed similarly to rats receiving neither MCAO nor IH (MCAOIH vs. Sham, escape latency, *p* = 0.99; swimming distance, *p* = 0.98) and to rats receiving IH intervention without MCAO (MCAOIH vs. NorIH, escape latency, *p* = 0.32; swimming distance, *p* = 0.07; Figure 1B–1C). These findings indicate that spatial learning impairments observed following brain ischemia are reversed by post-ischemia IH intervention.

Spatial memory was measured in a probe test conducted 1 hr after the final training trial on Day 14. During this test the platform was removed and time spent in each quadrant of the MWM was recorded. Consistent with the above findings, the MCAO group did not show a preference for the target quadrant (zone IV) whereas the Sham group did (*p*<0.05; Figure 1D). Moreover, the MCAOIH group spent significantly more time in the target quadrant than did the MCAO group (*p*<0.05) and performed similarly to the Sham group (*p = *0.998) and the NorIH group (*p = *0.73; Figure 1D). These findings suggest that spatial memory impairments observed following brain ischemia are reversed following post-ischemia IH intervention.

IH did not seem to produce a beneficial effect on spatial learning and memory in the absence of MCAO, as indicated by the similar performance of the NorIH group and the Sham group on all measures (escape latency, *p = *0.35; swimming distance, *p = *0.34; probe test, *p = *0.63; Figure 1B–1D). Thus, whereas mild IH intervention reverses spatial learning and memory impairments associated with ischemia, it does not appear to have any effect on learning and memory performance in normal rats.

### Neurogenesis induced by post-ischemia IH is necessary for spatial memory recovery

**Figure 2:**
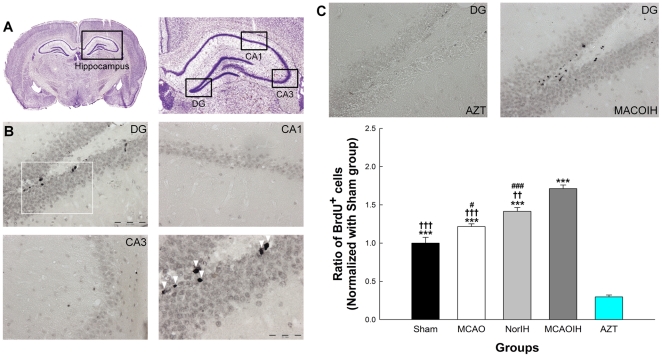
Post-ischemia IH intervention induced hippocampal neurogenesis. (A) Schematic depicting hippocampal subregions of interest. (B) BrdU^+^ cells in each hippocampal subregion (bar, 100 µm). The lower right image is a 40× magnification of the region boxed in white (bar, 50 µm). BrdU^+^ cells are indicated by arrows. BrdU^+^ cells were expressed primarily in the DG. (C, top) BrdU staining in the AZT group as compared to the MCAOIH group. (C, bottom) Quantification of BrdU staining. *p<0.05, **p<0.01, ***p<0.001, compared to the AZT group. ††*p*<0.01, †††*p*<0.001, compared to the MCAOIH group. #*p*<0.05, ###*p*<0.001, compared to the Sham group.

Because mild, transient hypoxia can induce neurogenesis and improve memory [14], we speculated that IH might ameliorate memory deficits observed following ischemia by stimulating the birth of new neurons. To test this hypothesis we injected rats with bromodeoxyuridine (BrdU), a marker of proliferating cells, on Days 8 and 14 and sacrificed them after they completed the MWM probe test on Day 14 (Figure 1A). We then used standard immunohistochemical techniques to stain for BrdU and counted BrdU^+^ neurons in the hippocampus to quantify neurogenesis (Figure 2B). Rats receiving IH treatment following ischemia (MCAOIH group), which as we described above showed a reversal of ischemia-induced spatial learning and memory impairments (Figure 1B-1D), exhibited a marked increase in neurogenesis as compared to the Sham group (*p*<0.001), MCAO group (*p*<0.001), and NorIH group (*p*<0.01; Figure 2C). These findings suggest that post-ischemia IH induces neurogenesis and that this effect may be associated with memory recovery. To more definitively establish whether neurogenesis is a mediator of IH-induced spatial learning and memory recovery, we evaluated spatial learning and memory and neurogenesis in the AZT group, which was exposed to MCAO, IH, and the neurogenesis-disrupting compound 3′-azido-deoxythymidine (3′-AZT). Interestingly, the AZT group showed significantly decreased neurogenesis as compared to all other groups (all *p*<0.001; Figure 2C) and exhibited significant impairments in spatial learning and memory as compared to the Sham, NorIH, and MCAOIH groups (all *p*<0.001; Figure 1B-1D). Collectively, these findings suggest that neurogenesis induced by post-ischemia IH is necessary for the reversal of ischemia-induced spatial learning and memory impairments.

However, the MCAO group also exhibited significantly increased neurogenesis as compared to the Sham group (*p*<0.05), even though the MCAO group showed deficits in spatial learning and memory (Figure 1B-1D and 2C). Furthermore, across all groups, the extent of BrdU staining did not correlate significantly with spatial memory performance (the difference of escape latency between Day 14 and 8 over Day 8 escape latency) in the MWM test (*r = *-0.29, *p = *0.09). Thus, neurogenesis can not fully account for IH-induced memory improvement following ischemia.

### Newborn cells appearing following post-ischemia IH exhibit activity related to memory recovery, as indicated by expression of c-Fos

**Figure 3:**
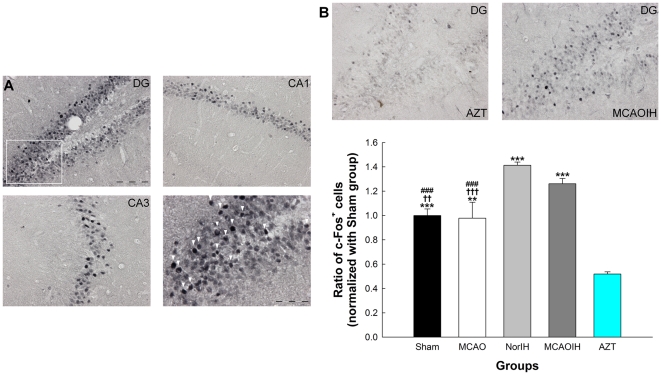
Post-ischemia IH intervention enhanced hippocampal cellular activity as indicated by c-Fos expression. (A) c-Fos staining in each hippocampal subregion (bar, 100 µm). The lower right image is a 40× magnification of the region boxed in white (bar, 50 µm). c-Fos^+^ cells are indicated by arrows. (B, top) c-Fos staining in the AZT group as compared to the MCAOIH group. (B, bottom) Quantification of c-Fos staining. **p<0.01, ***p<0.001, compared to the AZT group. ††*p<*0.01, †††*p<*0.001, compared to the MCAOIH group. ###*p<*0.001 compared to the NorIH group.

Because c-Fos is expressed in the hippocampus and is required for memory encoding and recall [15], [16], we speculated that IH might ameliorate ischemia-induced spatial learning and memory impairments by enhancing hippocampal c-Fos expression. To evaluate this hypothesis, we immunostained for c-Fos and counted the number of c-Fos^+^ hippocampal cells (Figure 3A). We observed a significant increase in the number of c-Fos^+^ cells in the NorIH group as compared to the Sham group (*p*<0.001; Figure 3B), suggesting that IH induces hippocampal c-Fos expression. Consistent with this, there was a significant increase in the number of c-Fos^+^ cells in the MCAOIH group as compared to the MCAO group (*p*<0.001) and the Sham group (*p*<0.01), but no difference in c-Fos expression between the MCAOIH group and the NorIH group (*p = *0.27; Figure 3B). The AZT group, by contrast, showed a significant decrease in the number of c-Fos^+^ cells as compared to the Sham (*p*<0.001), MCAO (*p*<0.01), NorIH (*p*<0.001) and MCAOIH groups (*p<*0.001; Figure 3B). Thus, IH intervention appears to induce hippocampal cell activity as indicated by c-Fos expression, but this effect is blocked by inhibiting neurogenesis.

**Figure 4:**
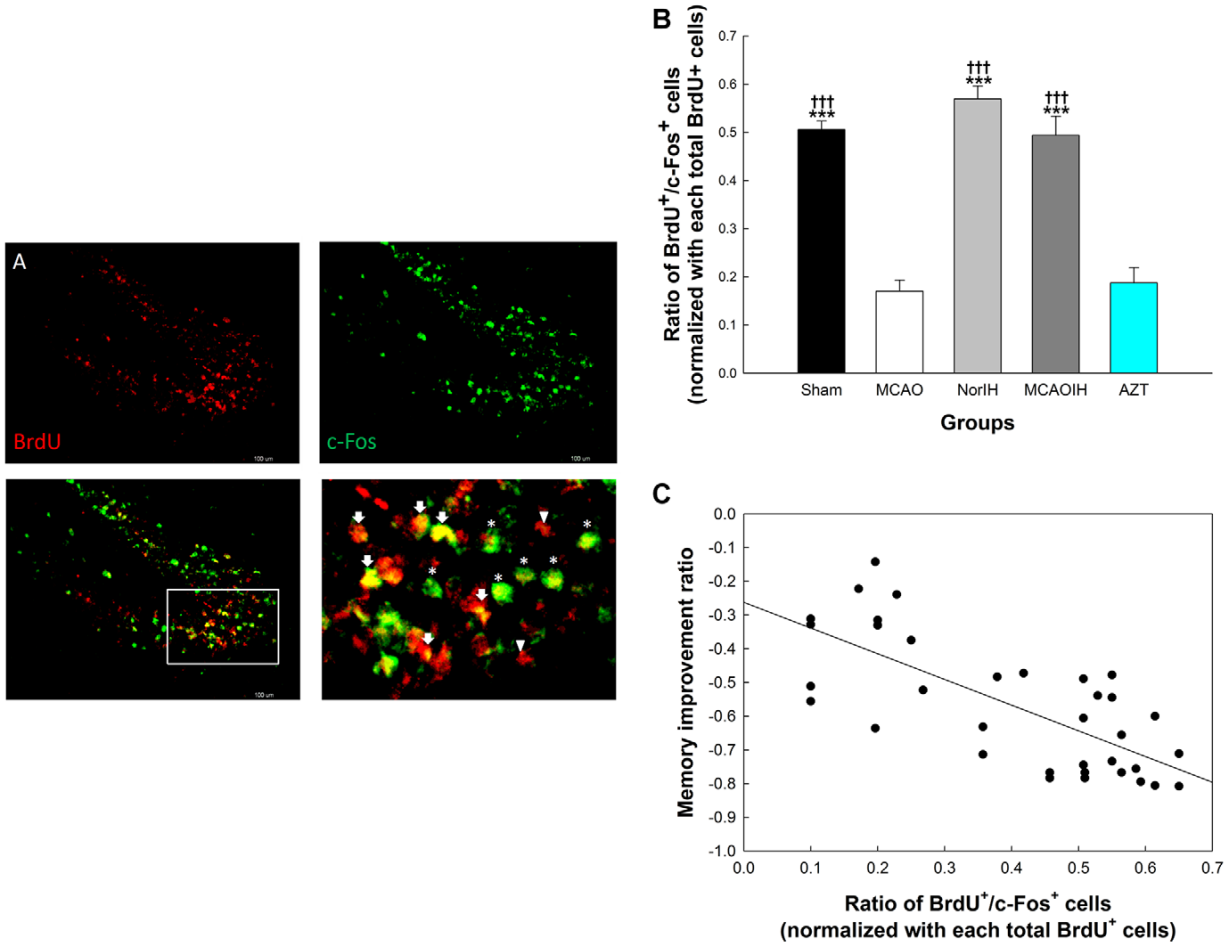
Post-ischemia IH intervention enhanced the activity of newborn cells, as indicated by c-Fos expression. Newborn cell activity correlated significantly with spatial memory improvement. (A) BrdU (red; upper left) and c-Fos (green; upper right) staining in the DG (bar, 100 µm). The lower right image is a 40× magnification of the region boxed in white (lower left). BrdU^−^/c-Fos^+^ cells are indicated by stars. BrdU^+^/c-Fos^+^ cells are indicated by arrows. BrdU^+^/c-Fos^−^ cells are indicated by arrowheads. (B) Quantification of BrdU^+^/c-Fos^+^ double-labeling. (C) Correlation of the number of BrdU^+^/c-Fos^+^ double-labeled cells with memory improvement ratios. ***p<0.001 compared to the AZT group. †††*p<*0.001 compared to the MCAO group.

Based on the expression patterns of BrdU and c-Fos, we hypothesized that the newborn (BrdU^+^) hippocampal cells exhibit activity related to memory recovery as indicated by c-Fos expression. To evaluate this possibility we performed double-label immunofluorescence staining for BrdU and c-Fos and counted the number of BrdU^+^/c-Fos^+^ cells (Figure 4A). We found that in the MCAO group there was a marked reduction in the number of double-labeled cells (0.17±0.02) as compared to the Sham group (0.51±0.02; *p*<0.001), suggesting that ischemia leads to a decrease in the activity of newborn cells (Figure 4B). By contrast, the NorIH group (0.57±0.03) and the MCAOIH group (0.49±0.04) both exhibited marked increases in the number of BrdU^+^/c-Fos^+^ cells as compared to the MCAO group (both *p*<0.001). There was no difference in the number of BrdU^+^/c-Fos^+^ cells in the NorIH group and MCAOIH group (*p* = 0.34), the NorIH group and the Sham group (*p* = 0.50), or the MCAOIH group and the Sham group (*p* = 0.998; Figure 4B). Collectively, these findings indicate that the ischemia-induced decrease in newborn cell activity is reversed by post-ischemia IH. In the AZT group there were fewer BrdU^+^/c-Fos^+^ cells (0.19±0.03) than in the Sham, NorIH, and MCAOIH groups (all *p<*0.001; Figure 4B). Interestingly, across all groups there was a significant correlation between the number of BrdU^+^/c-Fos^+^ cells and spatial memory performance (*r = *-0.73, *p<*0.001; Figure 4C), suggesting that the IH-induced recovery of newborn cell activity is necessary for memory recovery post-ischemia.

### Hippocampal MAPK phosphorylation and c-Fos expression are modulated similarly following IH intervention

**Figure 5:**
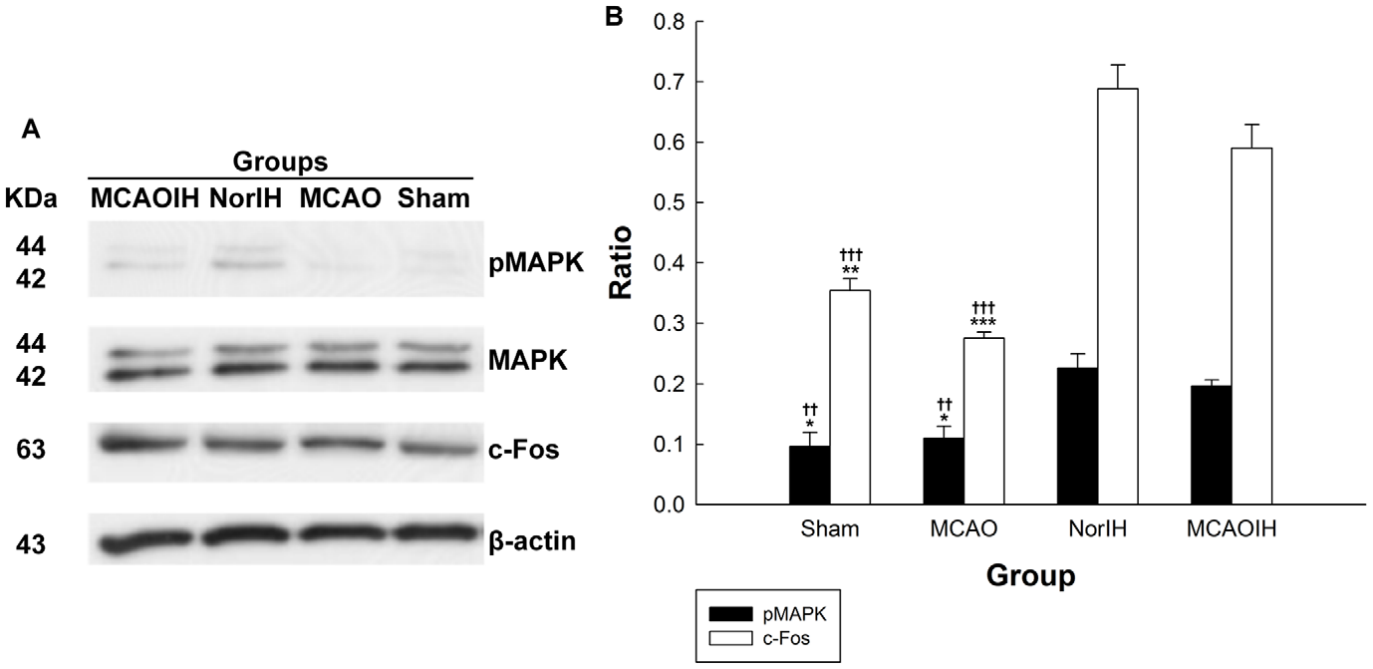
Post-ischemia IH intervention induced hippocampal c-Fos expression and MAPK phosphorylation. (A) Immunoblotting was used to detect hippocampal pMAPK, MAPK, and c-Fos expression. (B) Quantification of immunoblotting results. *p<0.05, **p<0.01, ***p<0.001, compared to the MCAOIH group. ††*p*<0.01, †††*p<*0.001, compared to the NorIH group.

c-Fos expression is regulated by phosphorylated mitogen-activated protein kinase (pMAPK), a second messenger implicated heavily in learning and memory [15], [17]. We speculated that hippocampal MAPK phosphorylation might increase following IH intervention. To test this we sacrificed rats in the Sham, MCAO, NorIH, and MCAOIH groups after they completed the MWM probe test on Day 14, then used immunoblotting to measure hippocampal pMAPK expression. We observed increased pMAPK expression in the NorIH group (*p*<0.01) and the MCAOIH group (*p*<0.05) relative to the Sham group (Figure 5A). pMAPK expression also was increased in the MCAOIH group relative to the MCAO group (*p*<0.05). There was no difference in pMAPK expression between the MCAOIH and NorIH groups (*p* = 0.73; Figure 5B). These findings suggest that IH intervention leads to upregulation of hippocampal MAPK phosphorylation.

Next, to determine whether c-Fos is regulated in a manner similar to pMAPK following IH intervention, we used a similar immunoblotting protocol to examine hippocampal c-Fos expression. Consistent with the pMAPK findings, we observed that c-Fos expression was increased markedly in the NorIH group (*p*<0.001) and in the MCAOIH (*p*<0.01) group as compared to the Sham group (Figure 5B). c-Fos expression also was increased in the MCAOIH group relative to the MCAO group (*p*<0.001). There was no difference in c-Fos expression between the MCAOIH and NorIH groups (*p* = 0.73; Figure 5B). Thus, c-Fos expression and MAPK phosphorylation follow a similar pattern, suggesting that MAPK phosphorylation may be related to c-Fos expression changes following IH intervention.

### Hippocampal hypoxia-inducible factor 1α (HIF-1α), c-Fos, and pMAPK expression levels increase with increasing numbers of IH interventions

**Figure 6:**
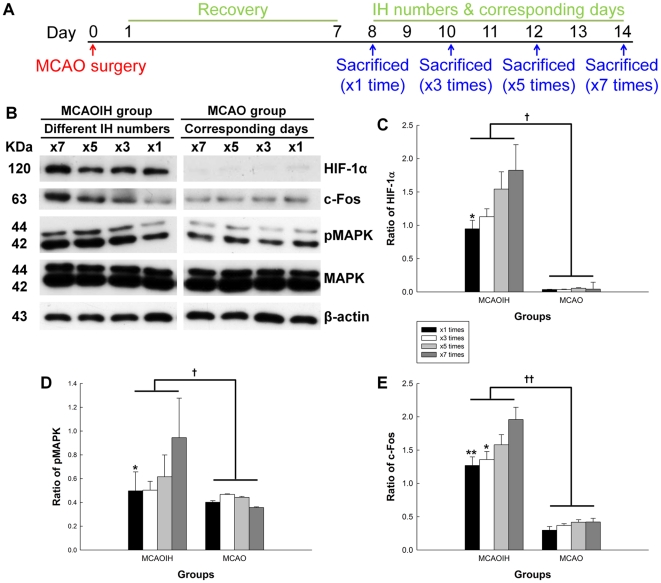
Hippocampal HIF-1α, c-Fos, and pMAPK expression increased gradually with increasing numbers of post-ischemia IH interventions. (A) Experimental design. Ischemic rats were exposed to differing numbers (1, 3, 5, or 7) of IH interventions or sham IH sessions. (B) Immunoblotting was used to detect hippocampal HIF-1α, pMAPK, MAPK, and c-Fos expression. (C–E) Quantification of immunoblotting results. *p<0.05, **p<0.01, compared to the MCAOIH ×7 group. †*p<*0.05, ††*p<*0.01, compared to the MCAO group.

Because hypoxia results in MAPK phosphorylation via HIF-1α [18], we hypothesized that HIF-1α might be a mediator of the hippocampal pMAPK and c-Fos expression changes that we observed following IH intervention. To examine this we exposed ischemic rats to differing numbers (1, 3, 5, or 7) of IH or sham IH exposures, sacrificed them immediately thereafter, and used immunoblotting to probe for hippocampal HIF-1α, c-Fos, and pMAPK (Figure 6A).

We observed that HIF-1α, c-Fos, and pMAPK expression increased in ischemic rats exposed to increasing numbers of IH interventions, but not in ischemic rats exposed to sham IH sessions (Figure 6B). HIF-1α expression was elevated significantly in ischemic rats that received seven IH interventions (MCAOIH ×7) as compared to ischemic rats that received only one IH intervention (MCAOIH ×1; *p*<0.05) or group effect of ischemic rats that received sham IH session(s) (MCAOIH vs. MCAO, *p<*0.05; Figure 6C). These findings suggest that HIF-1α expression is indeed regulated by IH and increases gradually with increasing numbers of IH interventions.

We also observed that pMAPK expression increased significantly in the MCAOIH ×7 group as compared to the MCAOIH ×1 group (*p*<0.05), and that c-Fos expression in the MCAOIH ×7 group increased significantly as compared to the MCAOIH ×3 group (*p*<0.05) and the MCAOIH ×1 group (*p*<0.01; Figure 6D-6E). pMAPK and c-Fos expression were also elevated significantly in the MCAOIH group relative to the MCAO group (MCAOIH vs. MCAO, *p<*0.05 and<0.01, respectively; Figure 6D-6E). These findings provide further evidence that pMAPK and c-Fos expression are modulated by IH, and suggest that their expression increases gradually with increasing numbers of IH interventions.

Interestingly, HIF-1α expression was highly correlated with c-Fos (*r* = 0.801, *p<*0.001) and pMAPK expression (*r* = 0.77, *p<*0.001). pMAPK and c-Fos expression were also correlated with one another (*r* = 0.72, *p<*0.01). Collectively, these findings are consistent with the hypothesis that HIF-1α acts as a mediator of hippocampal MAPK phosphorylation and c-Fos expression changes following IH intervention in ischemic rats.

## Discussion

We have shown that post-ischemia IH attenuates ischemia-induced spatial learning and memory deficits in rats. IH intervention induced hippocampal neurogenesis, but only those newborn cells that were activated (as indicated by expression of c-Fos) were involved in this memory enhancement. IH-induced c-Fos expression is likely mediated by pMAPK and HIF-1α.

Preconditioning IH intervention can attenuate memory impairments resulting from severe hypoxia or ischemia in rats [11], [12], although rapid, long term (12 hrs) exposure to hypoxic conditions (5.7% oxygen concentration) can have detrimental effects on memory by increasing oxidative stress and neuronal apoptosis [19]. In a previous study we found that exposure to 12% oxygen for 4 hrs/day for 7 successive days after brain ischemia can decrease infarct volume [20]._ENREF_15 The present study extended these findings by demonstrating that this same mild, post-ischemia IH intervention can attenuate ischemia-induced memory impairments.

Neurogenesis is implicated in learning and memory [9], [13], [21]. We found that IH intervention was associated not only with recovered memory in ischemic rats, but also with increased hippocampal neurogenesis. The effects of IH on memory and neurogenesis were diminished following disruption of neurogenesis via injections of 3′-AZT. However, across all groups, neurogenesis and memory performance were not correlated, suggesting that not all newborn cells contribute to memory recovery.

The transcription factor c-Fos is important in synaptic function and neuronal activity [16]_ENREF_26 as well as memory encoding and retrieval [15]. c-Fos expression is induced by ischemia, experience-dependent learning, and exposure to hypoxic conditions [2], [11]. Consistent with these observations, we found that c-Fos expression was induced in the hippocampus after IH intervention, suggesting that IH intervention leads to enhanced hippocampal cellular activity. We further noted that IH intervention following ischemia was associated with a significant increase not only in spatial memory performance but also in the number of BrdU^+^/c-Fos^+^ cells, and that these effects were blocked by attenuation of neurogenesis via 3′-AZT injection. It was previously reported that newborn neurons expressing c-Fos exert a long-term effect on DG function related to learning and memory [9]. This suggests that, in our study, only those newborn cells that expressed c-Fos following post-ischemia IH contributed to the spatial learning and memory recovery that we observed. Collectively, these findings suggest that post-ischemia IH intervention can stimulate hippocampal cell proliferation and enhance the activity of newborn cells, thereby contributing to memory recovery after ischemia.

Whereas another study reported that hypoxia can improve memory in normal animals [22], we found that normal rats showed no significant enhancement of either spatial learning and memory or newborn cell activity following IH. This inconsistency may reflect the use of a different learning and memory task, BrdU dose and injection protocol, and species. However, we did find that normal rats exhibited a marked increase in pMAPK and c-Fos expression following IH, suggesting that IH-induced MAPK phosphorylation and c-Fos expression can contribute to processes other than just memory enhancement. One possibility is that pMAPK mediates c-Fos stabilization, thereby contributing to cell proliferation [23]. In support of this possibility, we noted that there were significantly more newborn cells and significantly increased c-Fos expression in the NorIH and MCAOIH groups as compared to the Sham group.

A *c-fos* regulating element called the calcium response element (CaRE) is regulated by the Ras-Raf-MEK-MAPK/Erk pathway, such that MAPK/Erk activates its downstream kinase RSK/MSK to regulate the binding of the transcription factor CREB to CaRE [17]. In our study, increasing numbers of post-ischemia IH interventions led to gradual increases in MAPK phosphorylation, c-Fos expression, and HIF-Ια expression. Like c-Fos, HIF-Ια activity is regulated by MAPK/Erk [18]. We suggest that increasing IH intervention time results in increasing cellular activity via activation of MAPK, and that this is related to memory recovery after ischemia.

In conclusion, we observed that post-ischemia IH intervention rescued ischemia-induced spatial learning and memory impairments, likely by inducing hippocampal neurogenesis and c-Fos expression through mediators including pMAPK and HIF-1α.

## Materials and Methods

### Animals

Adult male Sprague-Dawley rats (8 weeks of age) were used. The rats were housed in groups of two in a temperature-controlled (22±1°C) vivarium with a 12 hr light: dark cycle (lights on at 0700 hrs), and had *ad libitum* access to food and water. All experimental protocols were approved by the Institutional Animal Care and Use Committee (IACUC) of National Yang-Ming University (IACUC approval No. 960306).

### Ischemia model: MCAO

The right middle cerebral artery was exposed through standard microsurgical techniques, and the right middle cerebral artery trunk was ligated above the rhinal fissure with 10-0 suture thread. Complete interruption of blood flow was confirmed with an operating microscope. Both common carotid arteries then were occluded with non-traumatic aneurysm clips for 1 hr, as described previously [20].

### Intermittent hypoxia (IH) intervention protocol

Rats were randomly assigned to the following groups: sham-MCAO without IH (Sham; n = 12), normal with IH (NorIH; n = 11), MCAO without IH (MCAO; n = 12), MCAO with IH (MCAOIH; n = 12), or MCAO with IH and 3′-AZT (AZT; n = 4), as shown in Figure 1A. Rats receiving MCAO were allowed to recover for 7 d prior to receiving IH or sham IH treatment, as in a previous study [20]. IH (12% oxygen) was administered for 4 hrs/d for 7 d in a hypoxia chamber (70×40×35 cm). Rats receiving sham IH sessions (i.e., Sham and MCAO groups) were placed in the hypoxia chamber and exposed to room air for 4 hrs/d for 7 d.

To determine the time course of the IH effects, separate MCAO groups of rats (n's = 4) received IH or sham IH treatment for 1, 3, 5, or 7 d and were sacrificed immediately thereafter (Figure 6A).

### Morris Water Maze (MWM)

On the same seven days as the IH or sham IH treatment (Days 8-14), all rats were trained on the MWM. The MWM apparatus consisted of a pool (180×60 cm) filled with water (23±2°C). Opaque curtains surrounded the maze and were affixed with high-contrast visual cues (an X, a triangle, a circle, and a square). The pool was divided arbitrarily into four equally-sized quadrants (called zones I, II, III, and IV). A 30-cm plexiglas platform (12 cm diameter) was submerged in zone IV such that its surface was 1 cm below the water surface. The room temperature was maintained at 25±1°C.

Rats were exposed to 3 training trials per day in which they swam freely until they located the hidden platform and remained on it for 30 s. Rats that did not locate the platform within 90 s were gently guided to it. After completing the last training trial on Day 14, rats rested for 1 hr in their home cages. They then received a probe test in which the platform was removed from the pool. During the probe test, the rats swam for 90 s and their behavior was recorded.

### Drug treatment: BrdU and 3′-AZT

Rats received intraperitoneal (i.p.) injections of BrdU (50 mg/kg, Sigma) on Days 8 and 14, and were sacrificed 1 hr after completing the MWM probe test on Day 14 (Figure 1A). Rats in the AZT group were also injected i.p. with 3′-AZT (100 mg/kg, Sigma), which disrupts neurogenesis [24], on Days 8–14 1 hr prior to IH intervention (Figure 1A). This group was included to evaluate whether neurogenesis is necessary for the ameliorating effect of post-ischemia IH on ischemia-induced learning and memory impairments.

### Immunohistochemistry

Rats were sacrificed after they completed the MWM probe test on Day 14. The brains were removed, fixed in 4% paraformaldehyde, and frozen at −80°C prior to being sectioned on a cryostat into 20- µm coronal sections ranging from −3.14 mm to −4.52 mm relative to bregma. Sections were mounted on slides (Thermo Scientific, superfrost). The slides were washed three times in PBS and incubated for 1 hr in blocking solution (3% normal serum, 1% serum albumin, 0.2% Triton X-100 in PBS). Antibodies were diluted to the appropriate concentration in the same solution. The primary antibodies used in these experiments were rabbit anti-c-Fos (1∶250; Santa Cruz) and rat anti-BrdU (1∶200; Accurate). Sections stained for BrdU were pretreated with 1N HCl for 30 min at 45°C and rinsed thoroughly in borate buffer (pH 8.6). The slides were next washed three times in PBS and incubated for 1 hr secondary antibodies. The secondary antibodies were biotin-conjugated rabbit (1∶500; Vector) and rat (1∶500; Vector). Visualization was performed with the ABC kit (Vector) and DAB-H_2_O_2_ chromogen system staining (Sigma). Sections were scanned using an Olympus BX-61 microscope. National Institutes of Health ImageJ software was used to quantify the staining in the following hippocampal subregions: CA1, CA3, and DG (Figure 2A).

### Immunofluorescence

Rats were sacrificed and brain sections were prepared as described above. Sections were incubated with rabbit anti-c-Fos antibody (1∶250; Santa Cruz), washed three times in PBS, and post-fixed in 4% paraformaldehyde for 20 min. They then were stained for BrdU (1∶200; Accurate). The slides were washed three times in PBS and incubated for 1 hr in PBS solution containing the fluorescence-conjugated secondary antibodies. The secondary antibodies were donkey anti-rat Alexa Fluor® 594 (1∶500; Invitrogen) and donkey anti-rabbit Alexa Fluor® 488 (1∶500; Invitrogen). Sections were mounted with Vectashield mounting media (Vector) and scanned using an Olympus BX-61 microscope. Staining was quantified using ImageJ software as described above.

### Immunoblotting

Rats were sacrificed after they completed the MWM probe test on Day 14. The brains were removed and the right hippocampus was dissected out and homogenized on ice in a lysis buffer [150 mM NaCl, 20 mM Tris (pH 7.5), 1 mM EDTA, 0.5% sodium deoxycholate, 0.1% SDS, and 1% nonidet P-40] containing a cocktail of a proteinase inhibitor (Roche) and a phosphatase inhibitor (Sigma). Samples were centrifuged at 12500 rpm at 4°C for 30 min and the supernatants were saved at −80°C for immunoblotting.

Protein concentrations were determined using the Bradford protein assay (Bio-Rad). Equal amounts of protein per sample (20 µg) were separated by electrophoresis on 8% and 10% Tris-glycine gels with 4% stacking gels, then were transferred to PVDF membranes. The membranes were blocked with 5% non-fat dry milk or 5% serum albumin for 1 hr. The primary antibodies were mouse anti-HIF-1α (1∶500; Novus Biologicals), rabbit anti-phospho-MAPK (1∶1000; Cell Signaling), rabbit anti-MAPK (1∶1000; Cell Signaling), rabbit anti-c-Fos (1∶200; Santa Cruz) and mouse anti-β-actin (1∶5000; Sigma-Aldrich). The secondary antibodies (all 1∶8000 diluted in blocking solution) were horseradish peroxidase conjugated anti-rabbit IgG and anti-mouse IgG (Chemicon). A horseradish peroxidase chemiluminescence kit with enhanced luminol and oxidizing reagents (Bio-Rad) was used to visualize the signal. Membranes were apposed to photographic film for detection of the bands, and the relative amount of each protein was determined using ImageQuant.

### Statistical analyses

The MWM acquisition data (escape latency and swimming distance) were analyzed using separate repeated measures analyses of variance (RM-ANOVAs). The MWM probe test data were analyzed using a one-way ANOVA. Group differences in c-Fos, BrdU, and BrdU/c-Fos immunohistochemical staining, and in protein expression levels in immunoblotting experiments, were analyzed using one-way ANOVAs. Data from the experiments examining changing HIF-1α, pMAPK, and c-Fos expression levels following increasing numbers of IH interventions were analyzed using separate two-way analyses of variance (two-way ANOVAs). Significant main effects were analyzed further using the Tukey post-hoc test. Correlations were analyzed using the Pearson correlation test. All data were expressed as mean ± SEM. Statistical significance was set at *p*<0.05.
